# Revealing the deposition of macrophytes transported offshore: Evidence of their long-distance dispersal and seasonal aggregation to the deep sea

**DOI:** 10.1038/s41598-019-39982-w

**Published:** 2019-03-12

**Authors:** Yutaka Kokubu, Eva Rothäusler, Jean-Baptiste Filippi, Eric D. H. Durieux, Teruhisa Komatsu

**Affiliations:** 1 Atmosphere and Ocean Research Institute, The University of Tokyo, 5-1-5, Kashiwanoha, Kashiwa-shi, Chiba, 277-8564 Japan; 2 Department of Biology, University of Turku, Turun yliopisto, FIN-20014, Turku, Finland; 3 SPE-UMR 6134 CNRS, University of Corsica Pasquale Paoli, BP 52, Corte, 20250 France; 4 UMS 3514 CNRS-UCPP Stella Mare Platform, University of Corsica Pasquale Paoli, Biguglia, 20620 France; 5 Present Address: Tokyo Metropolitan Research Institute for Environmental Protection, 1-7-5, Shinsuna, Koto-ku, Tokyo, 136-0075 Japan; 6 Present Address: Centro de Investigaciones Costeras-Universidad de Atacama (CIC-UDA), Avenida Copayapu 485, Copiapó, Atacama Chile

## Abstract

The role of coastal macrophyte beds as a carbon sink is under debate. Various studies have provided global estimates of the carbon sequestration and stocks of macrophyte beds; however, the final fate of macrophyte debris exported from coastal beds remains uncertain, and must be determined in order to fully clarify the role of coastal vegetation as a carbon sink. Here we conducted bottom-trawl surveys to investigate the extensive and seasonal aggregation of exported macrophytes on the continental shelf and slope seafloor (40–1,800 m). Sunken macrophytes showed a clear seasonal trend with highest biomasses in summer. This was mainly caused by the most collected macrophyte species *Sargassum horneri*. Furthermore, we used numerical simulations to verify the link between sea-surface hydrographic condition and seafloor distribution of sunken macrophytes. Our results showed that *S. horneri* accumulated beneath the Kuroshio Extension current, which is the western boundary current of the North Pacific subtropical gyre. Overall, floating macrophytes that became transported offshore by a stable sea-surface current, such as the western boundary current, constitute an organic carbon pathway from coastal waters to the deep sea. Our findings suggest that these buoyant macrophytes can act as a biological pump to enhance oceanic carbon sequestration.

## Introduction

Marine macrophyte beds of seaweeds and seagrasses are distributed in shallow coastal waters of temperate oceans worldwide. They have been recognized as one of the highest primary producers in the global carbon cycle^1^. The net primary production, which is the amount of CO_2_ fixed per unit area, is much higher in macrophyte beds than in phytoplankton blooms but is similar between macrophyte beds and terrestrial rain forests^2–4^.

Various studies have provided global estimates of carbon storage by macrophytes in the form of biomass and vegetated coastal sediments, which is known as “blue carbon”. Seagrass meadows, salt marshes, and mangroves have been recognized as the major contributor to this carbon storage^5–7^. Unlike these angiosperm-based habitats, seaweeds typically do not develop such organic-rich, and vegetated sediments^8^. Seaweeds grow on rocky hard substrata and when detached are neutrally buoyant and can float offshore as rafts (Supplementary Fig. S1), closely following the directions of the major currents^9–11^. When floating seaweeds lose buoyancy and sink to the seafloor, they transport biomass from the sea surface to the deep sea.

Hence, a vast quantity of seaweed biomass can become exported from the coastal euphotic waters to the offshore deep seafloor, where they significantly contribute to the vertical organic carbon flux in the ocean^12^. As a result, these allochthonous inputs of seaweed biomass may affect the deep-sea food webs^13–15^. Especially when they sink within and under the permanent thermocline i.e., the so-called mesopelagic ocean, the CO_2_ fixed by seaweeds becomes isolated from the atmosphere, causing a long-term carbon sequestration^12^, and thus acts as a biological pump. However, the fate of detached seaweeds lost to the deep sea remains unclear, and further research is needed to quantify these important contributions.

Several studies reported evidence of macrophyte aggregations on the offshore deep seafloor^16,17^. For instance, Wolff^18^ recovered fragments of seagrass and *Sargassum* from several stations off the east coast of the U.S. by dredging at bottom depths between 4,580 m and 8,330 m. Deep-sea photographic surveys using remotely operated vehicles in waters off the east and west coasts of the U.S. and in the Caribbean revealed a large number of seagrass blades and occasional massive deposits of *Macrocystis* and *Sargassum* on the seafloor at 100 m to 8,000 m depth^19–26^. A recent study by Takai *et al*.^27^ reported about species composition of macrophyte debris obtained by dredging and trawling at depths between 100 m and 400 m on the slopes of submarine valleys situated several kilometers off the Izu Peninsula of Japan.

However, those studies focused mainly on areas with concaved bathymetry, such as sea channels and submarine canyons, where macrophyte debris typically tends to accumulate. Even though the flat topographically simple bathymetry of the continental shelf is far broader than the concave areas, few studies have quantified the spatial distribution of sunken macrophytes at the continental shelf scale. Recently, Filbee-Dexter and Scheibling^28^ mapped for the first time sunken macrophytes in the continental shelf area along the Nova Scotia coastline in up to 140 m depth. This report is based on observations from epipelagic waters, thus the presence and distribution of floating macrophytes aggregating further offshore at deeper mesopelagic depths remains unknown.

**Figure 1:**
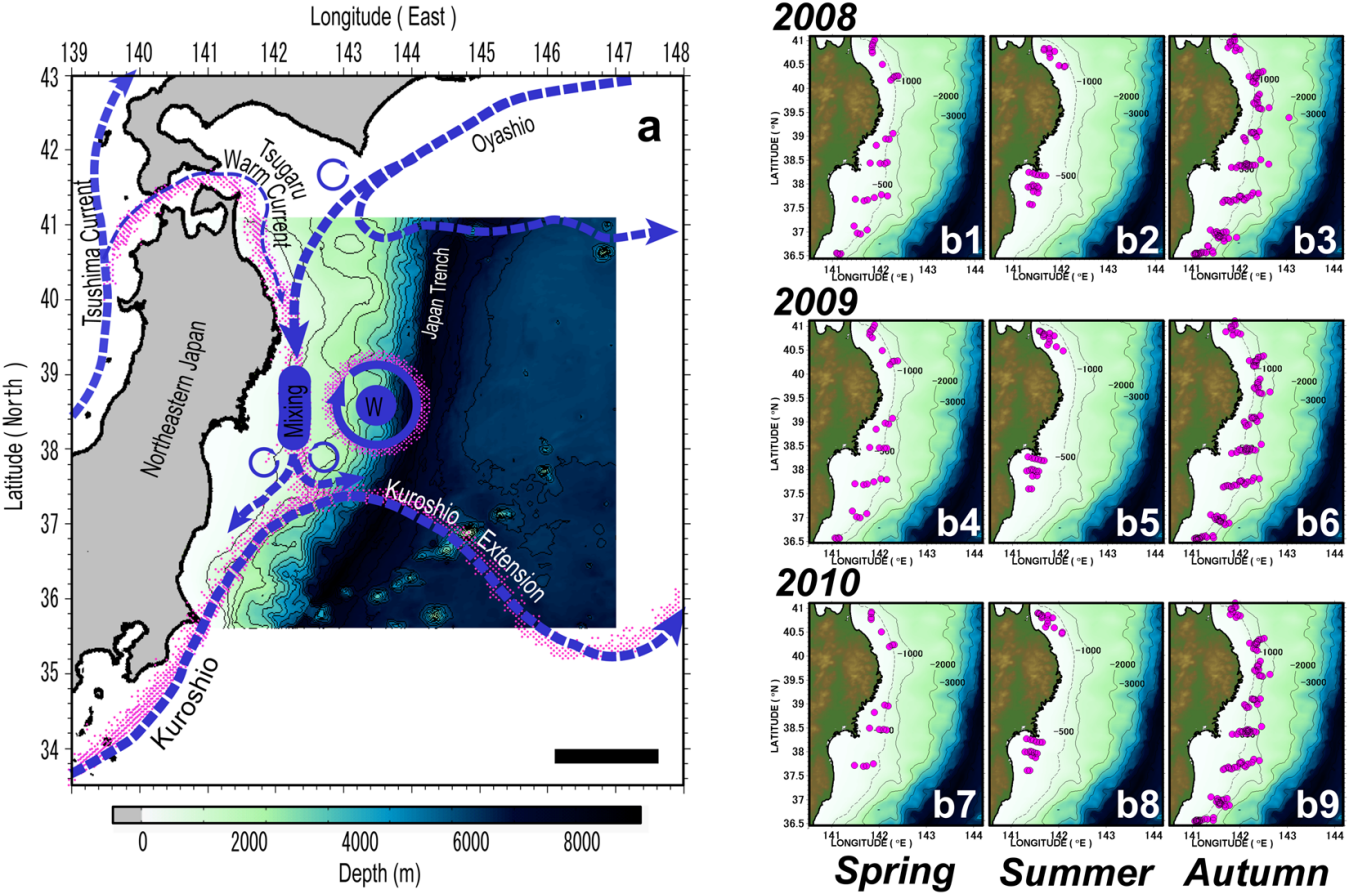
Study area superimposed on sea-surface hydrographic conditions (**a**). Purple areas close to the major sea-surface currents indicate where floating macrophytes were previously reported. The letter ‘W’ denotes a warm-water gyre that is isolated from the Kuroshio Extension current. Black scale bar = 200 km. (**b**) Location maps of bottom trawl stations (purple circles) in 2008 (upper panels: b1, b2, b3), 2009 (middle panels: b4, b5, b6) and 2010 (lower panels: b7, b8, b9), respectively for spring, summer, and autumn. The maps were generated using Matlab R2011b (https://www.mathworks.com/products/matlab).

In offshore sea-surface waters, floating items are generally concentrated in eddies and/or frontal systems. Floating rafts of buoyant macrophytes have also been reported to accumulate in the offshore fronts of sea-surface currents^29,30^. The sea-surface hydrographic conditions in the waters off the northeastern coast of Japan (this study) are governed by a southward intrusion of cold water from the Oyashio current and a northward intrusion of warm water from the Kuroshio Extension current^31,32^ (see Fig. 1a). In this region, floating macrophytes tend to concentrate along the coastal side of the Oyashio front, and around the northern periphery of the Kuroshio Extension front, but also in the outer margins of warm-water gyres that are isolated from the Kuroshio Extension current^33,34^ (Fig. 1a). Since macrophytes sink to the seafloor at a speed of >1,000 m per day^19,35^, their distribution on the seafloor might be influenced by their distribution on the sea surface. To investigate this hypothesis, we chose a survey area where the front of the Kuroshio Extension (western boundary current of the North Pacific subtropical gyre) is present throughout the year.

This study aimed to report the first direct evidence of sunken macrophytes on a continental-shelf scale bathymetry. Therefore, we conducted bottom-trawl surveys along ~500 km of the northeastern coast of Japan and up to 10–100 km offshore at depths between 40 m to 1,800 m. These surveys were done in spring, summer, and autumn over 3 years in order to test if there is a continuous supply of macrophytes to the seafloor. In addition, we used one of the most frequently collected seaweeds, namely *Sargassum horneri* (Turner) C. Agardh, to test whether sea-surface hydrographic conditions are linked to its distribution on the seafloor. We conducted two numerical experiments, the first one to identify the coastal origin of the sunken *S. horneri*, and the second one to identify areas where their chance to sink was highest.

## Results

### Macrophytes and their seasonal occurrence, biomass, and distribution

Two seagrass species, 12 *Sargassum* species (order of Fucales), and 3 species of the order Laminariales were collected during the bottom trawls within the 3 years. The collected seagrass was a mix of *Zostera marina* Linnaeus and *Phyllospadix iwatensis* Makino.

**Figure 2:**
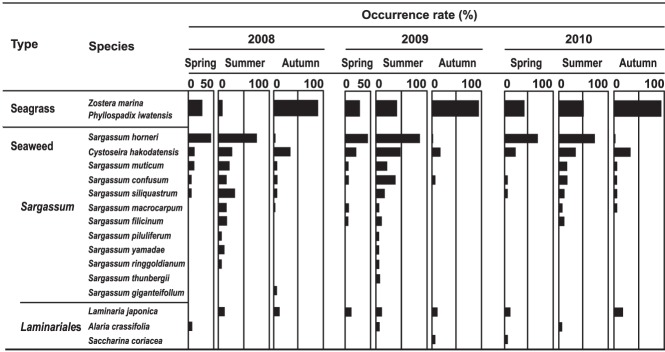
Occurrence rate of the different macrophyte species. Length of each horizontal bar corresponds to the occurrence rate (%) of each species.

Within the three years, the occurrence rate of *Sargassum*, *S. horneri*, and seagrass significantly differed among seasons (ANOVA, P < 0.01, Supplementary Table S1, Fig. 2) but no differences were observed among years (ANOVA, P > 0.05). *Sargassum* showed the highest occurrence rate in summer (mean = 87%, SD = 5, Tukey’s test P < 0.05, Supplementary Table S1) compared to spring (mean = 53%, SD = 3) and autumn (mean = 31%, SD = 16). For *S. horneri* the occurrence rate was significantly higher in spring (mean = 47%, SD = 14, Tukey’s test P < 0.05) and summer (mean = 70%, SD = 8, Tukey’s test P < 0.01) than in autumn (mean = 1.3%, SD = 0.6). In contrast, seagrass had the lowest occurrence rate in spring (mean = 37%, SD = 18) and summer (mean = 32%, SD = 22) whereas it was highest in autumn (mean = 89%, SD = 6, Tukey’s test P < 0.05). The chi-square test of independence between season and year was significant (P < 0.05, Supplementary Table S1), indicating that the occurrence rate varies seasonally within each year.

**Figure 3:**
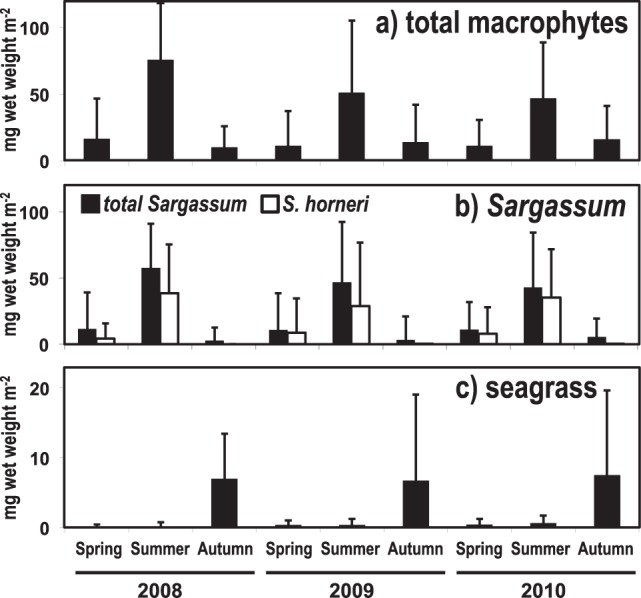
Seasonal variability of collected macrophyte biomass (mg wet weight m^−2^) within the three years. (**a**) Total macrophyte debris, (**b**) total *Sargassum* and *Sargassum horneri*, and (**c**) seagrass. Data are means ± SD.

Overall, average macrophyte biomass was highest in summer and lowest in spring and autumn (Fig. 3a). This converts to a carbon-based biomass of 0.8 ± 0.2 mg C m^−2^ (mean ± SD) in spring and autumn and of 3.5 ± 0.9 mg C m^−2^ in summer. This seasonality is mainly caused by the high biomass of *S. horneri* throughout all summers (Fig. 3b). Such as shown for the occurrence rate, the average biomass of seagrass within the three years was low in spring/summer but increased in autumn (Fig. 3c).

**Figure 4:**
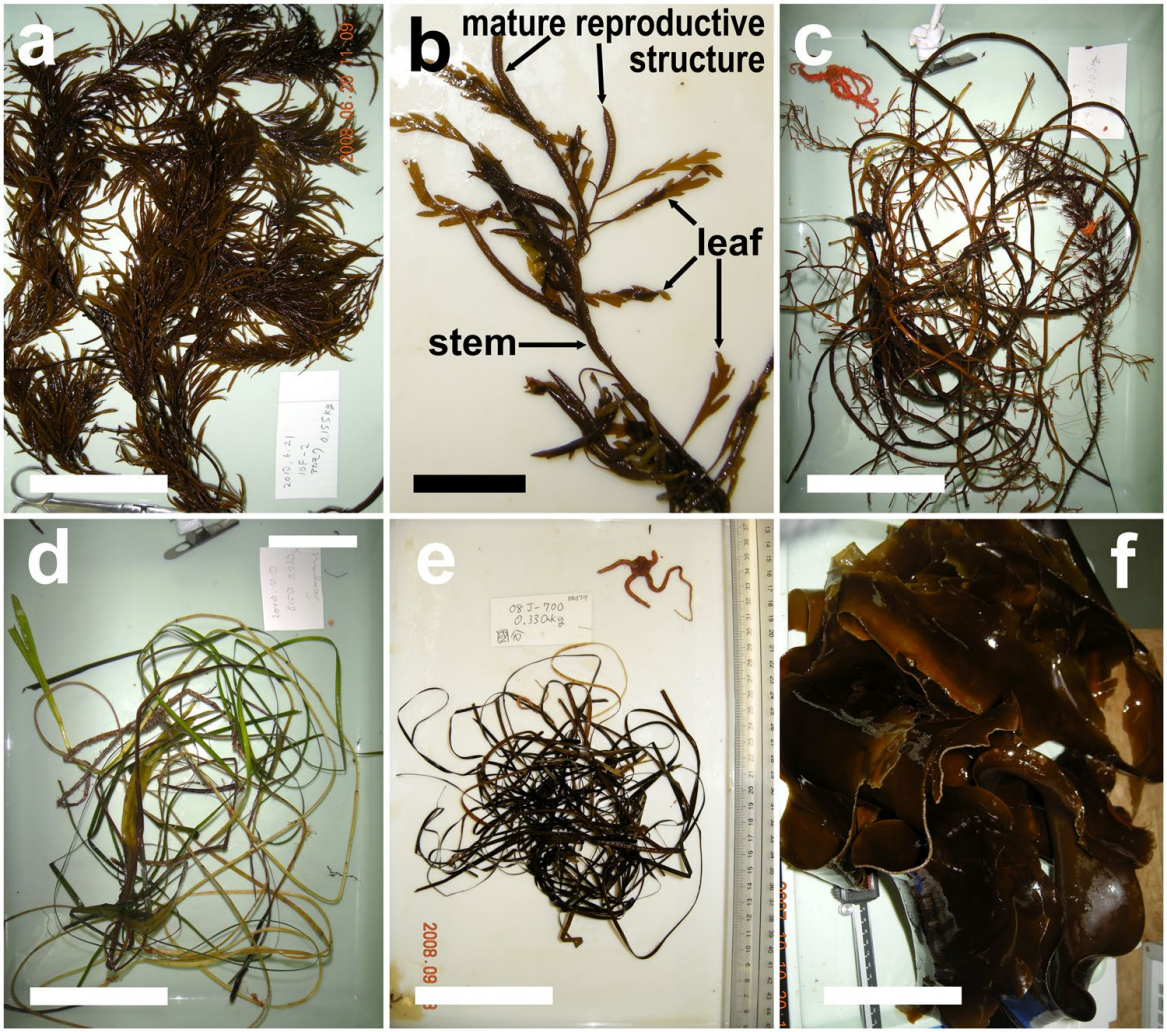
Examples of frequently collected macrophytes and their state of decomposition (freshness). (**a**) *Sargassum horneri* caught in summer, which was still in good condition. (**b**) *S. horneri* with intact receptacles and leaves attached to the stem. (**c**) Old *Sargassum* caught from 450 m depth in autumn. (**d**) Fresh seagrass caught in spring, and most of the blades had still a green color. (**e**) Old seagrass caught in summer. (**f**) *Laminaria japonica* aggregation collected at a depth of 350 m in autumn. White scale bar = 10 cm. Black scale bar = 2 cm.

We found that most of the *Sargassum* collected in spring and summer showed a good amount of leaves and reproductive structures (receptacles) that were in fresh conditions (Fig. 4a,b). In spring, old *Sargassum* was found in only one out of 45 samples (2%), whereas it was 11% (8/75) in summer. The opposite was true in autumn, where 98% of the samples (101/103) were represented by old *Sargassum* with only few leaves and reproductive structures. In addition, their stems were brittle and old (Fig. 4c). Similarly, only 4% of the seagrass samples collected in spring (1/28) were black and thus old, whereas the rest of the seagrass samples were green and relatively fresh (Fig. 4d). However, in summer 66% of the seagrass samples (19/29) were old, whereas it was 84% in autumn (249/297) (Fig. 4e).

**Figure 5:**
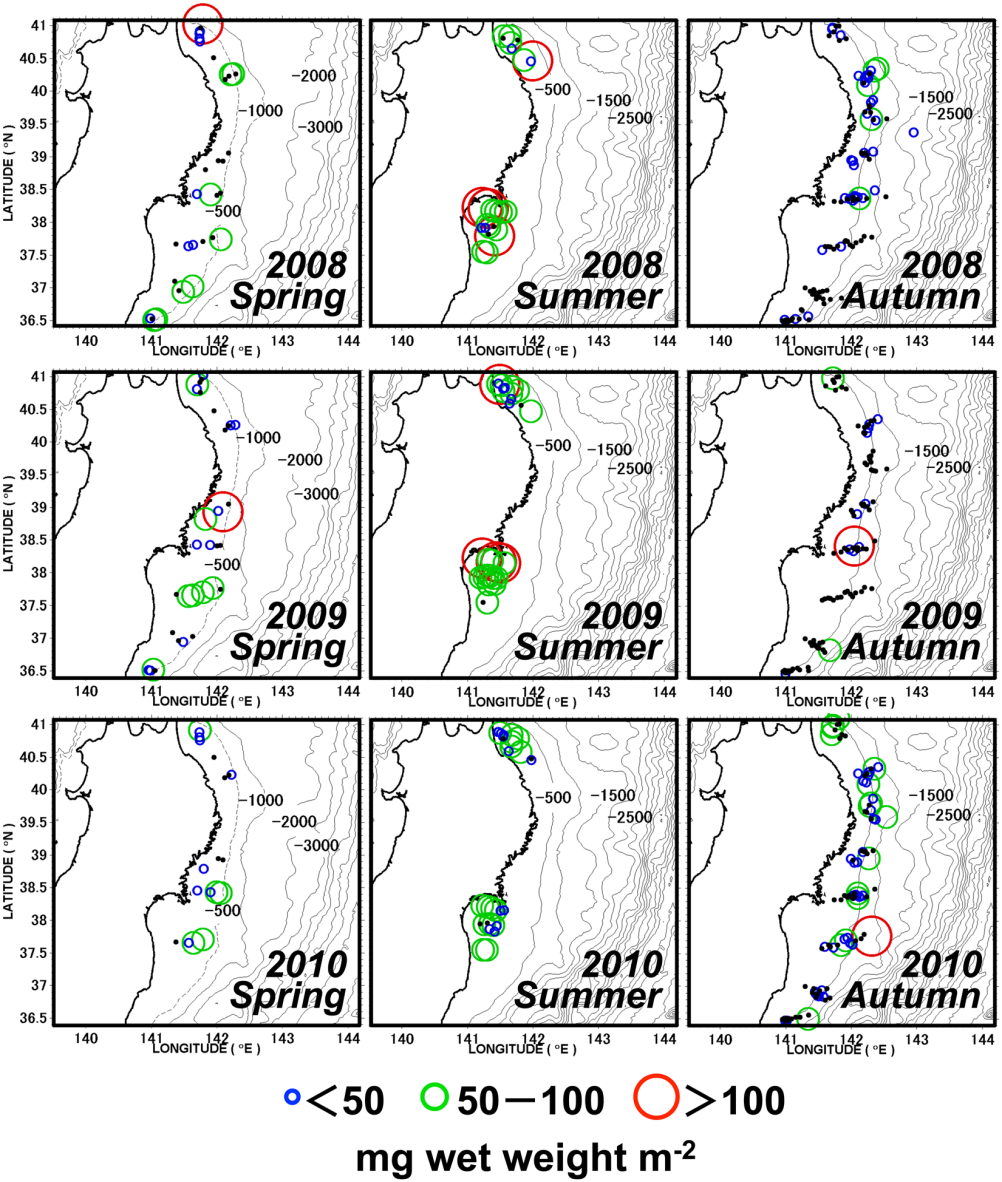
Distribution of *Sargassum* biomass on the seafloor. Black dots indicate stations without a catch. The maps were generated using Matlab R2011b (https://www.mathworks.com/products/matlab).

**Figure 6:**
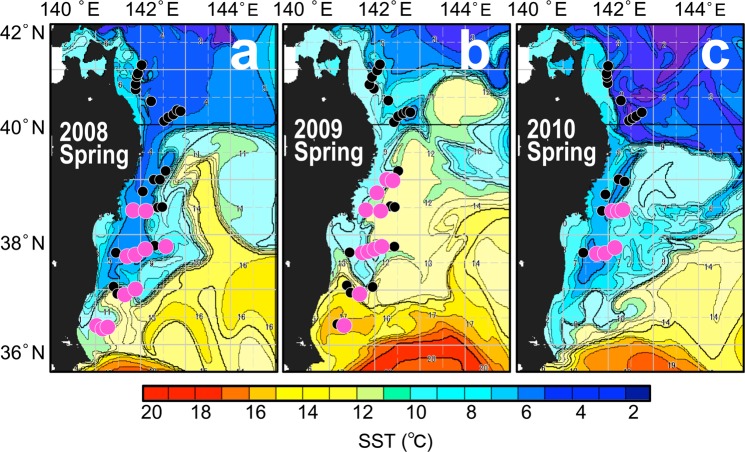
Distribution of *Sargassum horneri* debris (purple circles) in spring, which were superimposed on the sea-surface temperature satellite images from NOAA/AVHRR taken on April 15, 2008 (**a**), April 15, 2009 (**b**), and April 15, 2010 (**c**). Black dots indicate trawling stations without a catch. The maps were generated using Matlab R2011b (https://www.mathworks.com/products/matlab).

Within the entire survey area, *Sargassum* and seagrass were the most distributed macrophytes in all seasons (Figs 5 and S2, respectively), whereas the Laminariales mainly appeared in autumn, and then as large aggregates (Figs 4f and S3). None of the macrophyte biomasses (*Sargassum*, *S. horneri*, seagrass, and Laminariales) significantly correlated with the seafloor depth (Spearman’s rank correlation coefficient |ρ| < 0.4, P > 0.05 for all seasons throughout the 3 years). However, we found evidence that the distribution of *S. horneri* in spring was limited to the southern part (south of 39°N) of our study area (Fig. 6).

### Evaluation of origins, offshore-ward pathways, and accumulation zones

**Figure 7:**
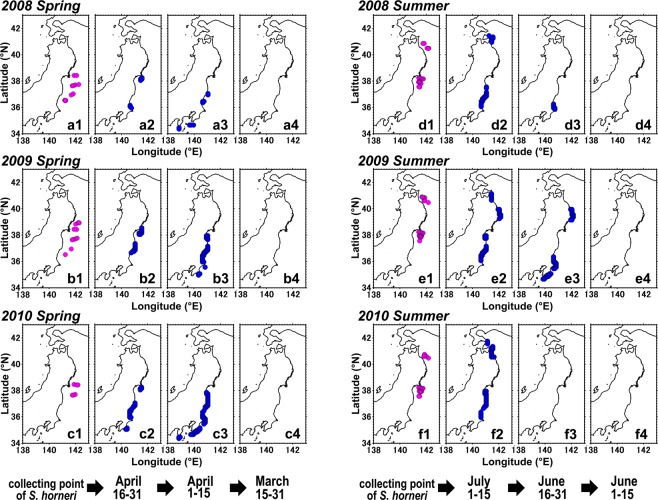
Numerically estimated origins of *Sargassum horneri* (blue dots) caught by bottom trawling in spring and summer of 2008 (upper panels: a, d), 2009 (middle panels: b, e) and 2010 (lower panels: c, f), respectively. Left (**1**) columns are trawl stations where *S. horneri* was collected in spring and summer, respectively. Columns (**2**), (**3**) and (**4**) are origins determined for 1–14 d, for 15–30 d, and for >30 d before the collection date of *S. horneri* debris, respectively. The maps were generated using Matlab R2011b (https://www.mathworks.com/products/matlab).

The numerical modelling revealed that the coastal origin of our collected *S. horneri* debris was along the Pacific coast of eastern Japan (Fig. 7a–c for spring, and **d**–**f** for summer). However, none of the *S. horneri* debris had its origins on the coast when the time was reversed for more than 30 d (Fig. 7a4,b4,c4 for spring, and **d4**, **e4**, **f4** for summer). Therefore, these simulation results indicate that the detachment of *S. horneri* occurred within one month before it was collected by our trawl net.

**Figure 8:**
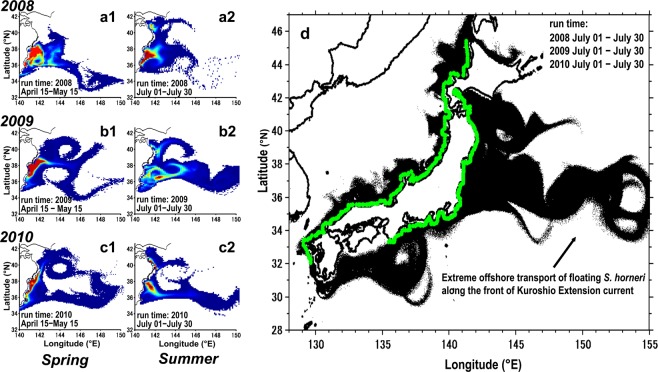
Accumulation zone of *Sargassum horneri* estimated by forward-in-time particle-tracking. One thousand particles were released from each estimated origin on April 15 (spring) and July 1 (summer) and traced for 30 d. Each day’s position of the particles was regarded as an accumulation zone of floating *S. horneri* in 2008 (upper panels: **a1**, **a2**), 2009 (middle panels: **b1**, **b2**) and 2010 (lower panels: **c1**, **c2**), respectively for spring and summer. Number of particles per unit area with high density is shown in red, and low density in blue. (**d**) Possible accumulation zones of floating *S. horneri* (black) transported from their previously reported benthic habitats along the Japanese coast (green). Particles were released from their initial position (green) on July 1 and traced for 30 d, in 2008, 2009 and 2010, respectively. Each day’s position of the particles for the three years were pooled and regarded as an accumulation zone of floating *S. horneri* in summer. The maps were generated using Matlab R2011b (https://www.mathworks.com/products/matlab).

The particle-tracking model simulated the offshore transport of *S. horneri* via sea surface currents, and thus gives indications about algal pathways and their main accumulation zones over the continental shelf seafloor (Fig. 8). For instance, the simulation showed that a large portion of the particles released from the estimated coastal origins, set for 1–30 days afloat, were demonstrated to distribute in waters between the Japanese coast and approximately 143 °E (shown in red in Fig. 8a–c). Since the *S. horneri* collection points (Fig. 7a1,b1,c1 for the spring, and d1,e1,f1 for the summer) were located inside the accumulation zone (Fig. 8a–c), the simulation results corresponded well to our bottom trawling results. Within 30 days of floating, the accumulation zone for *S. horneri* reached a longitude of 150° E (~800 km offshore) in the waters south of 38 °N latitude where the Kuroshio Extension current prevails. This extreme offshore transport was confirmed to occur on the Pacific side of northeastern Japan (Fig. 8d).

## Discussion

Previous surveys of floating macrophytes conducted close to our study sites showed that their maximum number was reached in early summer (May and June), with *S. horneri* as the most abundant species^36^. Those findings correspond well with the results found for our bottom-trawl surveys, where *S. horneri* was the most frequently collected species among the macrophyte debris in spring (April) and summer (June, July). Additionally, in both seasons, *S. horneri* dominated in the mass of macrophyte samples, but its maximum biomass was achieved in each of the three summers.

In our study, *Sargassum* individuals collected on the seafloor in spring and summer had receptacles, intact leaves and stems, whereas in autumn they were brittle and showed signs of decomposition. According to the study by Yatsuya^37^, detached *Sargassum* with receptacles stayed afloat for < 8 weeks. Hence, most of the *Sargassum* that float to the sea surface during their reproductive season (between spring and summer) probably sink to the seafloor before autumn. Furthermore, since detrital fragments of seaweeds gradually decompose^38–40^, it seems reasonable that the occurrence rate (see Fig. 2 and Supplementary Table S1) and biomass (see Fig. 3b) of *Sargassum* and *S. horneri* on the seafloor reached their main peaks when reproductive (spring/summer), whereas they sharply decreased in autumn. Based on these observations, it can be concluded that within our study area there is a stable and seasonal supply of newly produced *Sargassum* to the offshore seafloor.

In contrast, the biomass and occurrence rate of seagrass was low in spring and summer, whereas it increased in autumn throughout the 3 years. Seagrass is known to decompose much slower and less completely than seaweeds because of its high cellulose components^41,42^. In regard to the decomposition state (freshness) of the seagrass samples, it was clear that those collected in summer and autumn with a black color spent a longer period on the seafloor than those collected in spring (green color). Therefore it seems that even if the collected seagrass sank to the offshore seafloor soon after its maturity season during spring and summer, most of the blades did not decompose and thus accumulated until autumn.

The seafloor distribution of *S. horneri* in spring appeared to concentrate beneath the front of the Kuroshio Extension current, which passed through the southern margin of our study area, and around the outer margin of a warm-water gyre that was isolated from this current (Fig. 6). This implies that the spatial distribution of *S. horneri* debris on the seafloor is linked to the sea-surface frontal system. According to our numerical simulation results, the exportation trajectories of floating *S. horneri* from their estimated origin to the offshore areas occurred via a narrow stream along the path of the Kuroshio Extension current (Fig. 8a–c). This narrow stream was also responsible for the extreme offshore transport of *S. horneri* on the Pacific side of northeastern Japan (Fig. 8d). The accumulation zone for *S. horneri* when traced for 30 days extended up to 800 km offshore. Therefore our results show evidence that the *S. horneri* debris collected by us in spring and summer was transported offshore from the northeastern Japan archipelago via floating algae, and some got even caught in the Kuroshio Extension current. Algae caught within this current have the potential for a long-range export but also become entrained and thus sink below the current.

Due to its high persistence at the sea surface (8 weeks)^37^, we suggest that *S. horneri* can be found even further offshore from our survey area. For instance, at the northern periphery of the Kuroshio Extension front along 35.5–39°N latitude, Safran and Omori^33^ found floating macrophyte rafts at ~1,500 km offshore during May/June. Similarly, Kokita and Omori^34^ reported that in May floating macrophytes distributed at about 300 km offshore around the route of the Kuroshio Extension front, which is located parallel to the 35.6°N latitude. Thus we expect that floating macrophytes, mostly *S. horneri*, concentrate along the Kuroshio Extension front, and may sink to the seafloor at even greater depths.

Since most of the *Sargassum* and seagrass species detach from the bottom substratum after reaching the highest biomass during their reproductive seasons in spring and summer^43–47^, their detrital contribution to the ocean floor varies seasonally. *Sargassum* debris probably disappears totally from the seafloor before the next autumn due to decomposition and/or consumption by detritivores. Non-buoyant Laminariales such as found herein, were collected as large aggregates at a few sampling points. It is assumed that these non-buoyant algae become transported offshore along the submarine slope via gravity and bottom currents. Smaller detrital particles degrade at a faster rate than larger aggregates because the latter ones have a higher surface area relative to their volume for microbial colonization^38^. Thus some of them get gradually decomposed or consumed while drifting along the seafloor, whereas larger aggregations of Laminariales debris may endure these processes and have the chance to reach the mesopelagic depth. We speculate that these allochthonous inputs of seaweed-derived carbon are fueling the deep-sea food webs.

In contrast, the seagrass debris with its high and persistent cellulose components decomposes much more slowly than seaweeds^7^. Thus, even if the exported seagrass debris mainly sinks to the offshore seafloor soon after spring, the blades may endure decomposition and accumulate until autumn but are completely decomposed and/or buried in sediments before the next year’s spring. Decay of seagrass detritus is speculated to occur either via burial in the coastal habitat or by transport to the deep sea along the submarine slope.

The calculated carbon-based biomass of macrophytes collected herein was within a range of 0.6–4.4 mg C m^−2^ (if the carbon content ratio of macrophytes was 6% of their wet weight, see Material and Methods). A study done by Harrold *et al*.^23^, using photographs from remotely operated vehicles, estimated carbon based biomasses at the continental shelf (87 m to 357 m depth) of ~0.1–10 mg C m^−2^, and at the submarine canyon (153 m to 454 m depth) of ~100–1,000 mg C m^−2^. Except for the submarine canyon, their results resembled similar carbon-based biomasses as obtained by us. This similarity between the temperate east and west Pacific implies that macrophytes with a long-range transport, assisted by strong offshore-ward sea surface currents, have a higher chance to become exported from their site of origin toward a broader area of the continental shelf.

When macrophytes reach the seafloor of the mesopelagic ocean, the CO_2_ fixed by photosynthesis becomes precluded from the exchange with the atmosphere over extended timescales, even after being remineralized^12^. Consequently, these macrophytes act as a biological pump and promote long-term carbon sequestration in the deep sea. Especially, buoyant macrophytes with a long-range export might be the most effective type for capturing and transferring atmospheric CO_2_ to great depths, thus contributing to blue carbon.

Future studies should expand their survey areas further offshore in order to better understand the role of macrophyte beds as a biological pump in the ocean carbon cycle. These survey areas can be chosen with reference to the findings obtained from numerical simulation models as performed in this study. Since the organic carbon source on the offshore seafloor is generally thought to be particles that originate mainly from phytoplankton, these studies will lead to a fuller understanding of the ocean carbon cycle and its role as a sink for anthropogenic CO_2_.

## Materials and Methods

### Study site and collection of sunken macrophytes

Our bottom trawl surveys were conducted in waters off the coast of northeastern Japan where macrophyte debris, including seaweed (order of Fucales and Laminariales) and seagrass were collected. The trawling stations were systematically set along several latitudinal transects and vertical to the isobaths in order to verify the spatiotemporal characteristics of the sunken macrophytes (Fig. 1b). These surveys were conducted in spring (April), summer (June, July), and autumn (October, November) of 2008, 2009 and 2010. The total numbers of trawling stations in spring, summer and autumn were 34, 25, and 113 in 2008, 33, 30, and 109 in 2009, and 19, 31, and 109 in 2010, respectively. Within the three years, we used two research vessels, namely T/V Tanshu-maru (Kasumi Fisheries Senior High School) and R/V Wakataka-maru (Japan Fisheries Research and Education Agency).

In order to sample macrophyte debris, we used a bottom-trawl net with a mouth opening of about 19 m (horizontal width) (Supplementary Fig. S4). The net was rigged with bridles and otter windows. The mesh size was 8 mm. We towed the net for 30 min at a speed of 2.5–3.5 knots at each station. To prevent tearing of the net, the towing duration was sometimes adjusted according to the conditions of bottom obstacles and the volume of catches. The net was deployed at bottom depths of 120–550 m in spring, 40–200 m in summer, and 120–1,800 m in autumn.

### Onboard treatment and freshness evaluation of sunken macrophytes

After each sampling, catches of macrophyte debris were immediately checked using a 10 by 10 mm mesh sieve. To ensure that our bottom samples did not include floating macrophytes, caught during the ascent or descent of the net, we put the samples in a water tank. Samples with positive buoyancy were excluded. Thereafter, we identified the macrophyte species and recorded their wet weight.

The state of macrophyte decomposition (freshness) was determined by visual observation based on morphological characteristics for *Sargassum*, and on color for seagrass. In *Sargassum*, the gas vesicles, reproductive structure (receptacle), and leaves (Fig. 4b) are among the first parts to decay^37,47,48^. Thus we defined the sample as old if air vesicles, reproductive structures, and leaves were almost completely lost from the stem (Fig. 4c). For seagrass, the blade color is a good aging indicator because it markedly changes from green to black according to the state of its decaying process^49^. Therefore, seagrass samples with black colored blades were defined as old (Fig. 4e).

### Biomass estimation and statistical analysis

The biomass of macrophyte debris per unit area (mg wet weight m^−2^) at each trawl station was calculated by the following procedure. The mouth width of the bottom trawl net during the trawling operation was measured with an otter recorder (Furuno, CN-22A) 10 minutes before the ascent of the net from the seafloor. The average mouth width of the net was measured to be 19 ± 6 m (mean ± SD, *N* = 503). A net-mounted probe attached to the head rope was used to determine the towing duration at each trawl station; i.e., the period from when the net reached the bottom until it left the bottom. Each vessel was equipped with a GPS to determine the towing distance of the bottom trawl net. Using the mouth width of the bottom-trawl net and the towing distance, we calculated the seafloor area swept by the bottom-trawl net. The mean swept area at each trawl station through the 3-year survey was 25,200 ± 9,150 m^2^ (mean ± SD, *N = *503).

According to the bottom-trawl net swept area, we calculated the macrophyte biomass *D* (mg wet weight m^−2^) at each trawl station as follows,

1 $$D=W/A\cdot E$$

where *W* is the wet weight of the macrophyte debris caught by the net, and *A* is the swept area. We divided the wet weight of the catch (*W*) by the area swept in each tow (*A*), and then corrected by the catch efficiency (*E*) of the bottom trawl net.

The catch efficiency *E* of the bottom trawl net used in this study was previously estimated for macrophyte debris by Kokubu *et al*.^50^ (for more details see description in Supplementary Information). They obtained for two independently conducted experiments a catch efficiency of 0.19 and 0.15. Therefore, we herein calculated macrophyte biomass with an average catch efficiency of 0.17. The obtained macrophyte biomass was then roughly converted to carbon quantities. Since the carbon content ratio of marine macrophytes is assumed to be generally about 6%^51–53^ of their wet weight, we used this ratio to calculate the carbon-based amounts (mg C m^−2^). However, due to the uncertainty of the macrophyte decomposition states, this estimation of carbon quantities are likely to be in the upper range of the actual values.

In order to examine whether the macrophyte biomass depended on the depth, a Spearman’s rank correlation coefficient was examined for all seasons over the 3-years. In addition, we examined the seasonal variability of dominant macrophyte species in the study area by determining their rate of occurrence for each season (i.e., the percentage of each species observed among all trawling stations). Analysis of variance (ANOVA) followed by the post hoc Tukey’s multiple comparison tests were conducted to determine whether the occurrence rate of macrophyte species (*Sargassum*, *S. horneri*, and seagrass) differed significantly between seasons and years. The chi-square test of independence was used to determine the interaction between season and year. Statistical analyses were performed using the open-source software R (Ver. 3.4.3, CRAN, The Comprehensive R Archive Network), and the software BullCurve for Excel (Ver. 3.4.3, Social Survey Research Information Co., Ltd.).

### Numerical estimation of coastal origins and offshore accumulation zones

We used a two-way particle tracking method (two-way PTM) to determine the coastal origin of the bottom collected *S. horneri*. This method was applied earlier by Isobe *et al*.^54^ to specify the origins of floating marine debris in the East China Sea. Authors confirmed that the two-way PTM provides estimates with higher reliability than other conventional numerical methods.

Two components were required to use the two-way PTM origin estimation algorism: a particle tracking simulation code to simulate the floating macrophyte trajectory and a sea-surface velocity forcing data. We used a floating macrophyte particle tracking simulator developed by Filippi *et al*.^55^. The basic attributes of this simulator are described in the Supplementary Information. We used the sea-surface velocity forcing data of the OFES (Ocean General Circulation Model for the earth simulator) calculated by JAMSTEC (the Japan Agency for Marine-Earth Science and Technology). The spatial resolution of the OFES velocity data was 0.1° (zonal and meridian) with a temporal frequency of 3 days^56^. The OFES outputs around Japan in 2008, 2009 and 2010 was used to simulate the floating macrophyte trajectories. We applied the horizontal diffusivity value of 100 m^2^ s^−1^ observed from the Argos satellite tracking buoys off the Pacific coast of northeastern Japan^57^ for our calculation of the particle trajectory. The practical procedure of two-way PTM conducted along with the above-mentioned simulator and the OFES velocity-forcing data is described in the Supplementary Information.

We conducted an additional forward tracking simulation to identify the main accumulation zones of *S. horneri* over the continental shelf seafloor. This was done for the three springs and summers. In the simulation, one thousand particles were released from each of the estimated coastal origins. The particles were released on April 15 for the three springs and July 1 for the three summers, respectively, and traced for 30 days. The particle position of each day was regarded as a possible accumulation zone of floating *S. horneri* transported from its estimated origins.

A similar simulation was applied to identify possible accumulation zones of *S. horneri* exported from their entire distribution along the Japanese coast. Therefore, we released the forward-tracking particles from regions where benthic *S. horneri* has been previously reported by Yoshida^47^ and Komatsu *et al*.^58^. This was done only for the three summers (July 1), and the particle position of each day was pooled and regarded as the summer accumulation zone of *S. horneri*.

## Supplementary information

Supplementary Information
