# Supplementary material for: Revealing the deposition of macrophytes transported offshore: Evidence of their long-distance dispersal and seasonal aggregation to the deep sea
Source: Sci Rep. 2019 Mar 12;9:4331. doi: 10.1038/s41598-019-39982-w (PMC6411727; doi:10.1038/s41598-019-39982-w)
Supplement: Supplementary file 1 — Supplementary Information [file 41598_2019_39982_MOESM1_ESM.docx]

**Revealing the deposition of macrophytes transported offshore: Evidence of their long-distance dispersal and seasonal aggregation to the deep sea**

Yutaka KOKUBU ^1,+,*^, Eva ROTHÄUSLER ^2,++^, Jean-Baptiste FILIPPI ^3^, Eric D. H. DURIEUX ^3,4^, Teruhisa KOMATSU ^1^

^1^ Atmosphere and Ocean Research Institute, The University of Tokyo, 5-1-5, Kashiwanoha, Kashiwa, Chiba 277-8564, Japan

^2^ Department of Biology, University of Turku, Turun yliopisto, Turku, Finland

^3^ SPE-UMR 6134 CNRS, University of Corsica Pasquale Paoli, BP 52, Corte 20250, France

^4^ UMS 3514 CNRS-UCPP Stella Mare Platform, University of Corsica Pasquale Paoli, Biguglia 20620, France

^+^ Present address: Tokyo Metropolitan Research Institute for Environmental Protection, 1-7-5, Shinsuna, Koto, Tokyo 136-0075, Japan

^++^ Present address: Centro de Investigaciones Costeras - Universidad de Atacama (CIC - UDA), Avenida Copayapu 485, Copiapó, Atacama, Chile

^*^ Corresponding author: kokubu-y@tokyokankyo.jp


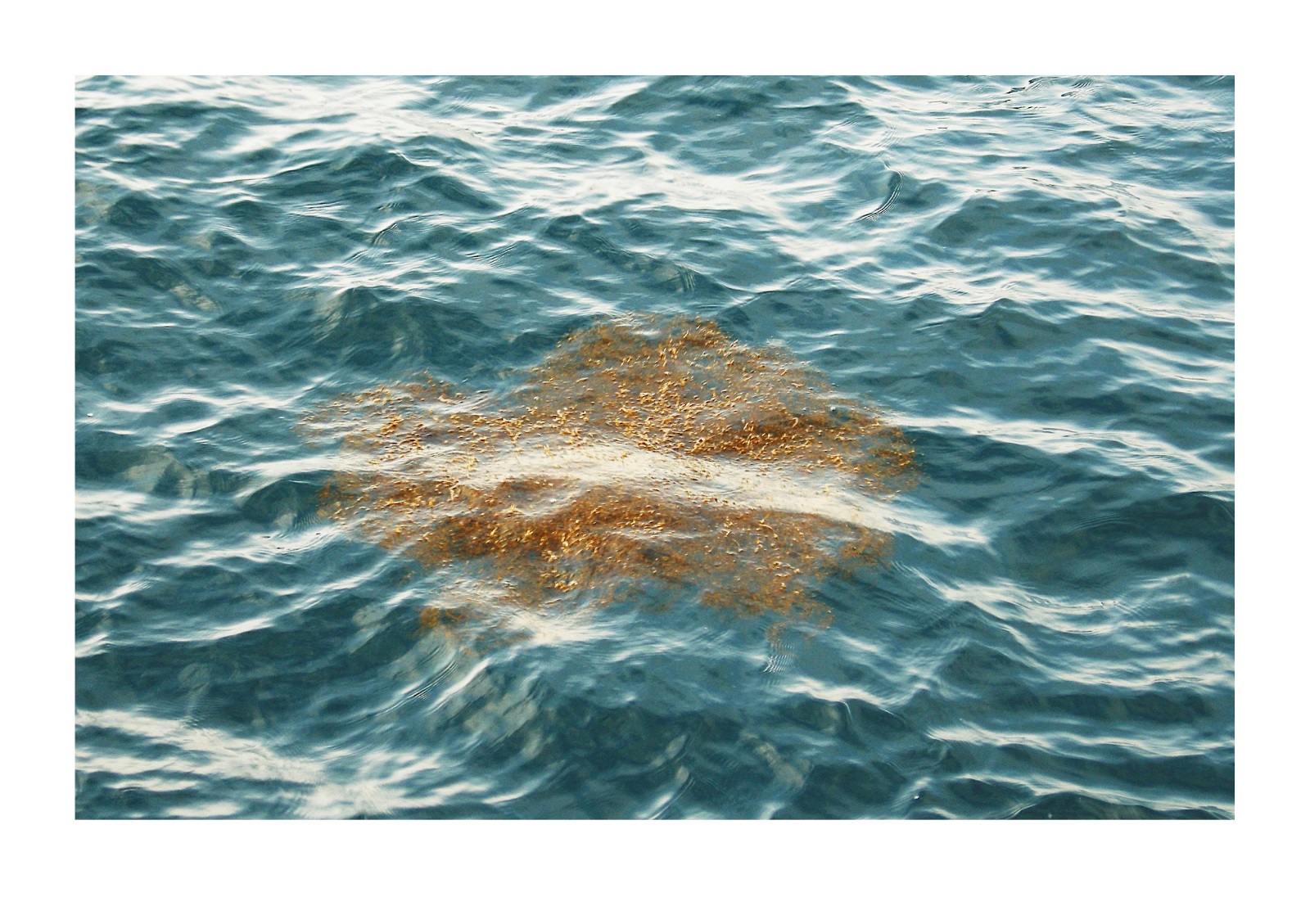


**Supplementary Fig. S1.** A raft of *Sargassum* floating in our survey area off the Pacific side of northeastern Japan. The diameter of the raft represents approximately 5 m.

**Supplementary Table S1.** Statistical results, comparing the occurrence rate of macrophytes (*Sargassum*, *S. horneri*, and seagrass) between season and year. The occurrence rate of each macrophyte differed significantly among seasons (ANOVA, P < 0.01) but not among years (ANOVA, P > 0.05). The difference between each season was isolated with the post hoc Tukey’s test (asterisks denote significant difference; * P < 0.05, ** P < 0.01). Chi-square test of independence indicates significant interaction between season and year (P < 0.05).


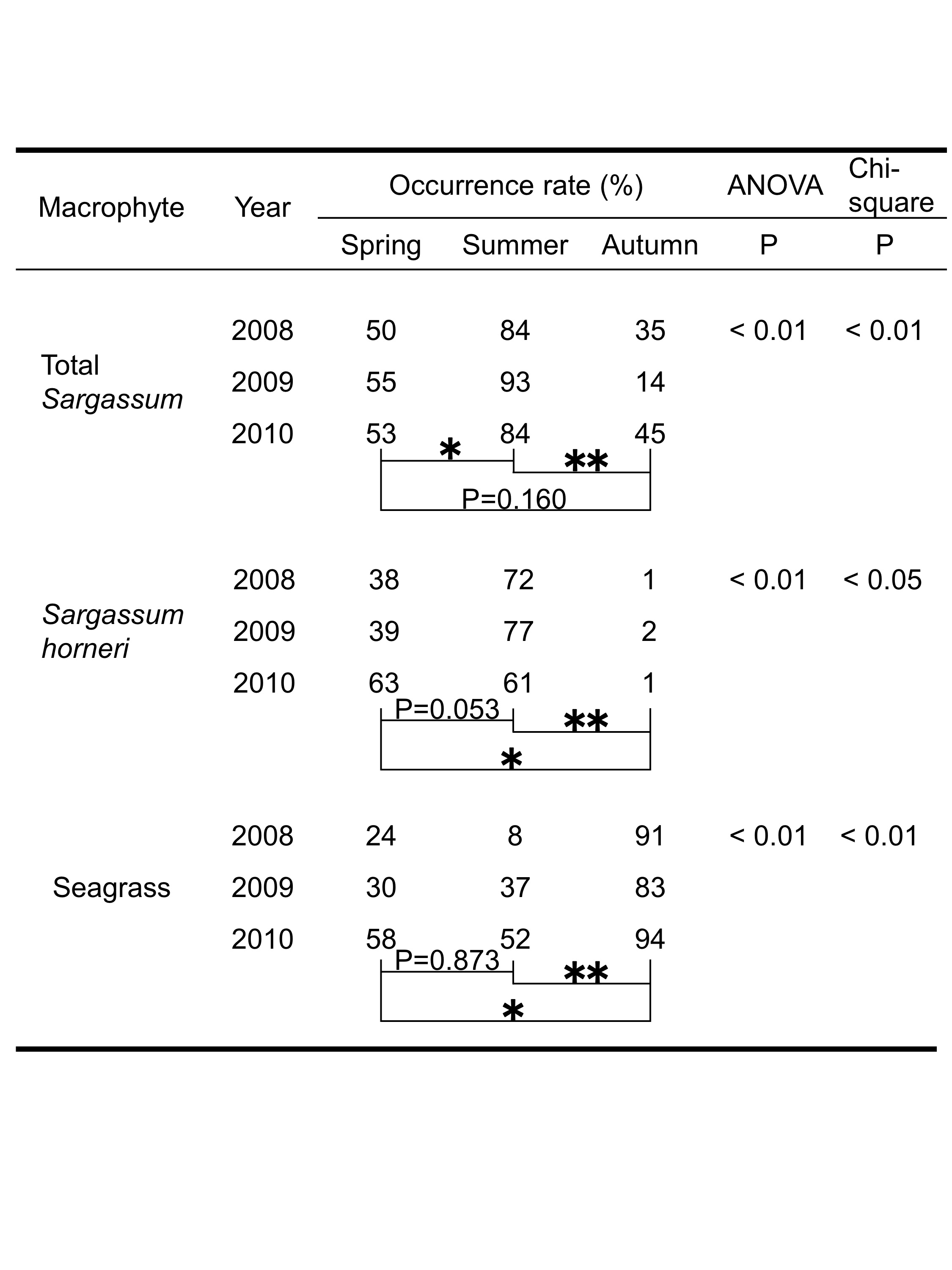


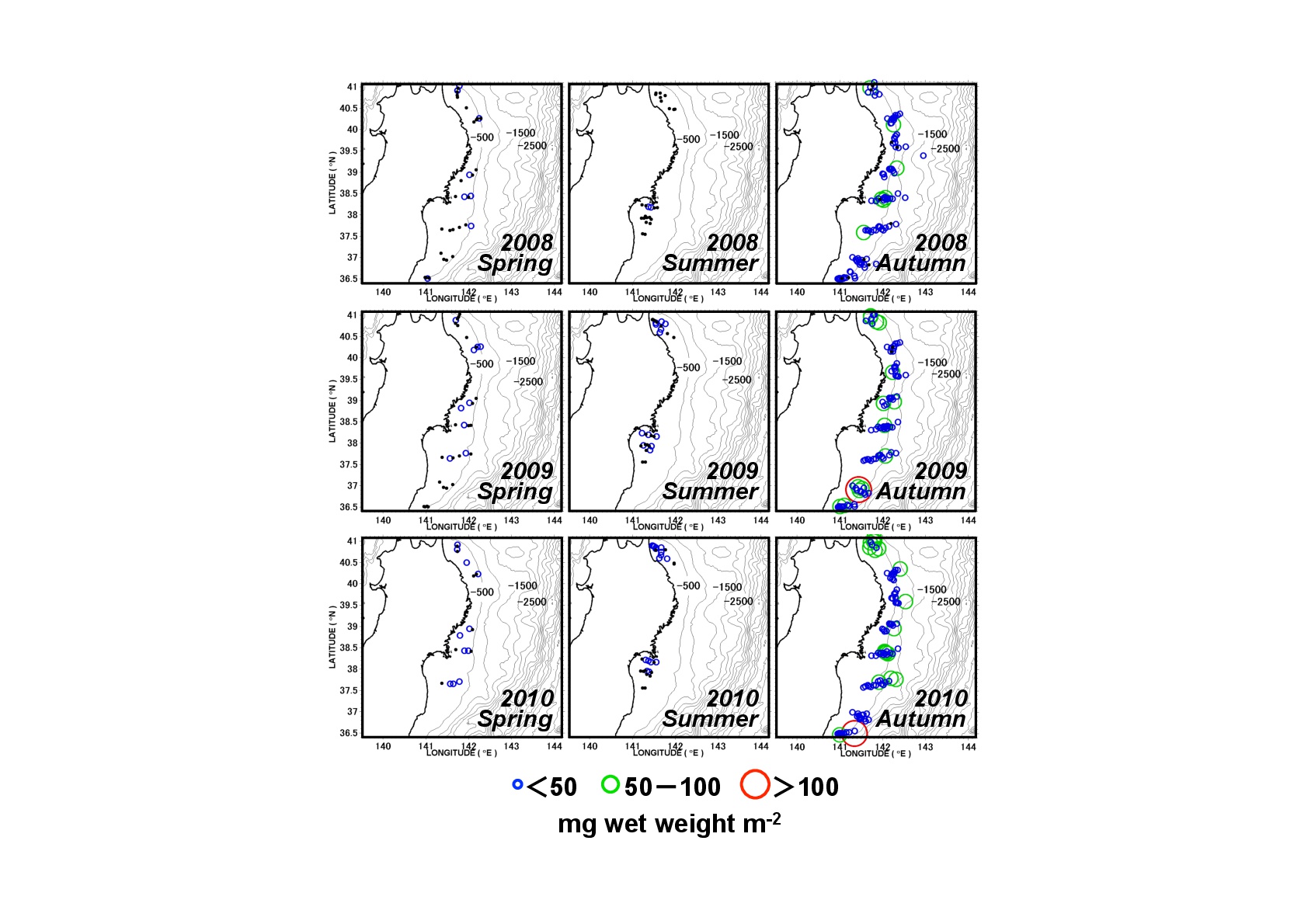


**Supplementary Fig. S2.** Distribution of seagrass biomass on the seafloor. Black dots indicate stations without a catch. The maps were generated using Matlab R2011b (https://www.mathworks.com/products/matlab).


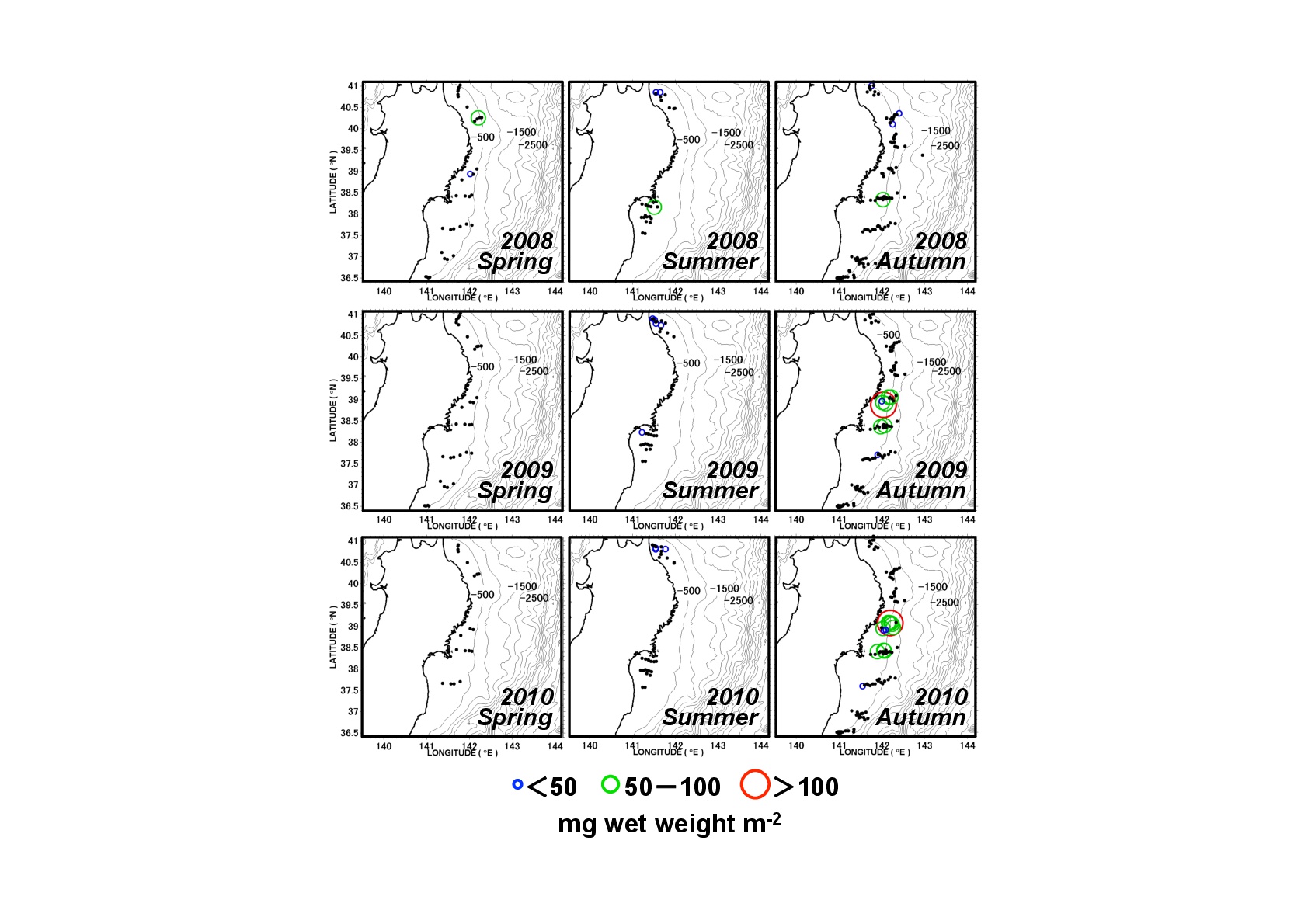


**Supplementary Fig. S3.** Distribution of Laminariales biomass on the seafloor. Black dots indicate stations without a catch. The maps were generated using Matlab R2011b (https://www.mathworks.com/products/matlab).


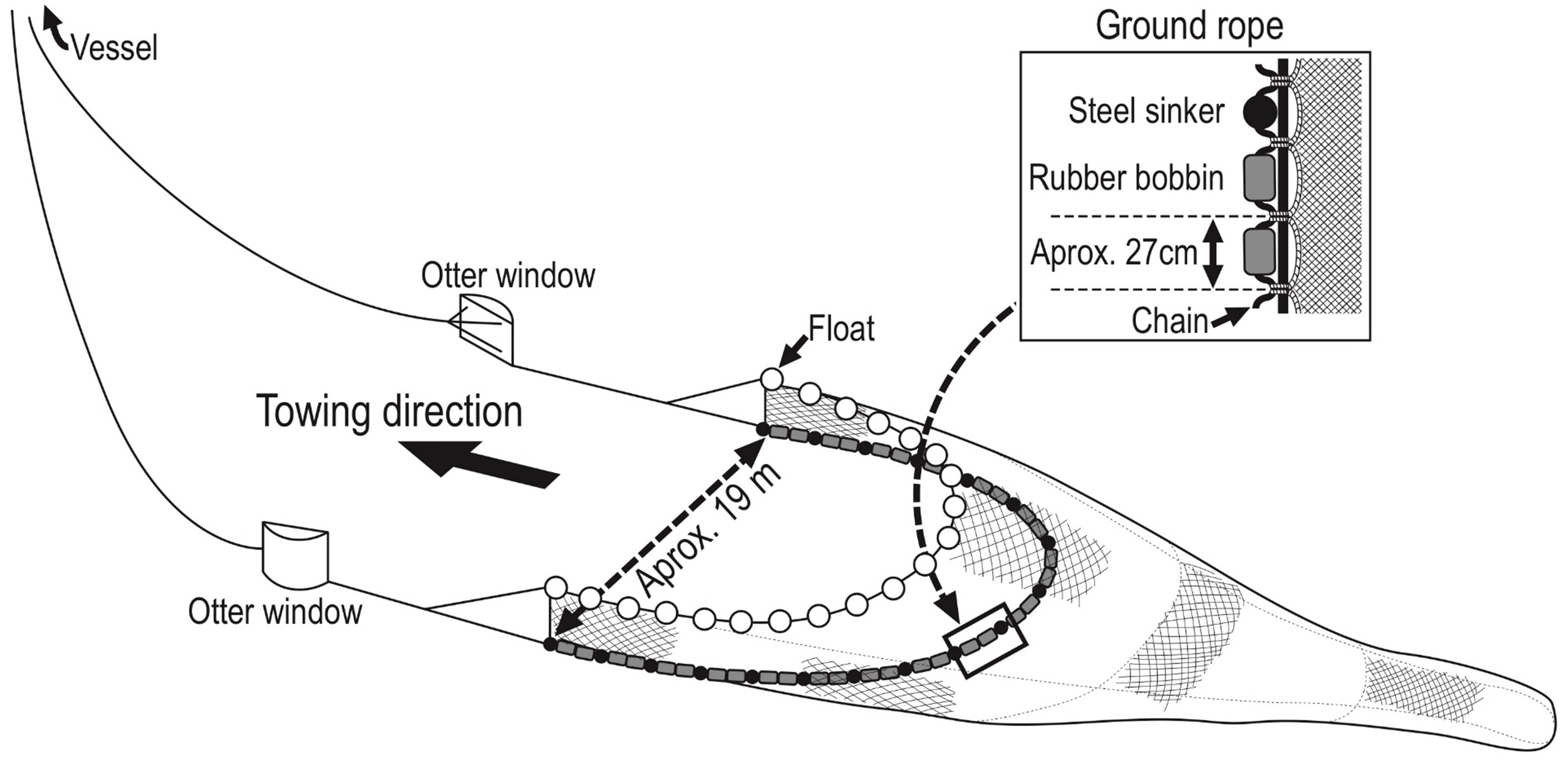


**Supplementary Fig. S4.** Configuration of the bottom trawl net used in this study and close-up of the ground rope at the entrance of the trawl net.

**Catch efficiency estimates of the bottom trawl net**

The catch efficiency of the bottom trawl net for macrophytes were examined by two procedures: a “Frame trawl net experiment” and the “Extra net experiment” (Kokubu *et al.*, 2012). Each of the procedures is detailed below.

Kokubu Y., Komatsu T., Ito M., Hattori T. & Narimatsu Y. Biomass of marine macrophyte debris on the ocean floor southeast of Hokkaido Island adjusted by experimental catch efficiency estimates. *La mer* **50**, 11–21 (2012).

*Frame trawl net experiment*

In order to simulate the actual bottom trawl net used in the field survey, an experimental frame trawl net was designed (Supplementary Fig. S5**a**). This experimental frame trawl net was equipped with the same ground rope as the actual bottom trawl net, which is illustrated in Supplementary Fig. S4. Four rubber bobbins and one steel sinker were attached to the frame trawl net ground rope. An underwater video camera (DMC-FT1, Panasonic Co.) was mounted on the center of the upper part of the frame trawl mouth. It was oriented to view the entire ground rope. The video images obtained were used to count the number of benthic obstacles entering the net mouth during the towing operation. *Sargassum horneri* was used for the catch efficiency experiment because it was the most frequently collected species among the macrophyte debris in this study. Experiments were performed in July 2009 with freshly harvested *Sargassum horneri* from Otsuchi Bay on the Pacific side of northeastern Japan. Fragments of freshly cropped *S. horneri* were prepared to sink toward the seafloor by removing their buoyant vesicles. Two hundred fragments of artificial *S. horneri* debris were prepared, each with a weight of 50 g (Supplementary Fig. S5, **b**). They were randomly placed in Funakoshi Bay (39.383 °N and 141.942 °E) at 15 m depth on a 20 m^2^ area with sandy seafloor. The experimental area was previously surveyed by side-scan sonar and confirmed to be a sandy bottom. This was done because the actual bottom trawl surveys were conducted on a sandy bathymetry. After *S. horneri* fragments were scattered on the bottom, the frame trawl net was towed by boat across the seafloor of the experimental area at a speed of 2.5 knots. The fragments collected with the frame trawl net were examined after each tow and weighed to determine their wet weights. The collected wet weights were defined as the *CW*. The number of fragments (*FN*) entering the net mouth during the trawling operation was counted using the obtained video image. From the obtained data, the catch efficiency *E* was defined as follows.

$E= \frac{CW}{FW・FN}$ ・・・・・・・・・・・・(s1)

*FW* is the wet weight of each *S. horneri* fragment (50 g). We obtained the catch efficiency *E* by dividing *CW* by the wet weight of the fragments passing the frame trawl mouth, which was calculated by multiplying *FW* by *FN* in equation (s1). We observed the total amounts of *S. horneri* fragments passing through the mouth of the frame trawl net on video images (Supplementary Fig. S5**c**). By comparing the total amount passing through the mouth of the frame trawl net with the total amount caught in the net as mentioned above, the catch efficiency was calculated. As a result, we obtained a catch efficiency of 0.19 (6.9 kg that passed the mouth and 1.3 kg captured).


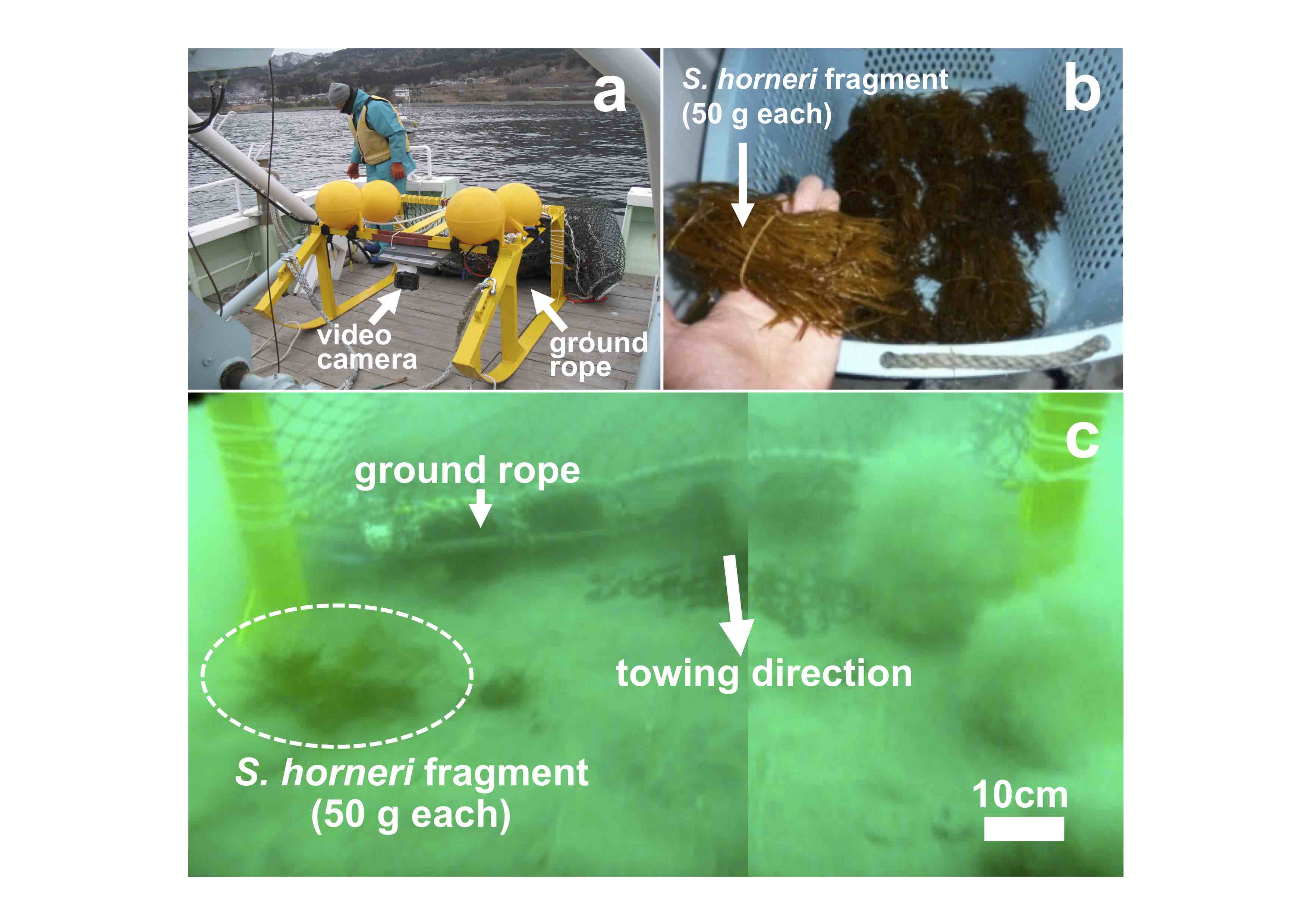


**Supplementary Fig. S5.** (**a**) Front view of the experimental frame trawl net used for the catch efficiency estimates of the bottom trawl net. (**b**) *S. horneri* used for the frame trawl net experiment. (**c**) The ground rope approaching *S. horneri* fragment during the “frame trawl net experiment” is indicated by the broken line ellipse. *Extra net experiment*

The second experiment used to estimate the catch efficiency of the bottom trawl net was different from the above-mentioned “frame trawl net experiment”. This experiment was carried out by covering the actual bottom trawl net in Fig. S4 with an experimental cover net. A schematic diagram of this net is depicted in Supplementary Fig. S6. Distinct from the inner actual bottom trawl net that was equipped with a regular ground rope, the outer experimental cover net of 8 mm mesh size was equipped with a chain ground rope in order to have better contact with the seafloor. Since macrophyte samples smaller than the 10 mm mesh sieve were not collected in our bottom trawling survey, the catch efficiency of the cover net was assumed to be 1.00. Thus, the catch efficiency *E* of the inner bottom trawl net was estimated by comparing the two weights of macrophyte debris caught by the inner actual bottom trawl net and the outer experimental cover net as follows.

$E= \frac{TW}{(TW+CW)}$ ・・・・・・・・・・・・(s2)

*TW* represents the wet weight of the macrophyte debris sample caught by the inner bottom trawl net, and *CW* represents the wet weight of the debris caught by the outer cover net. We performed the experiments at 23 stations between depths of 150 m and 450 m by T/V Tanshu-maru in April of 2010. The experimental net was towed for 30 minutes with a speed within the range of 2.5 to 3.5 knots at each trawling station. Fragments of *S. horneri* debris were caught in both the inner and outer nets at the same time in 4 of 23 tows (Supplementary Table S2). As a result, the catch efficiency for *S. horneri* was estimated to be 0.15 ± 0.07 (mean ± SD, *N* = 4).


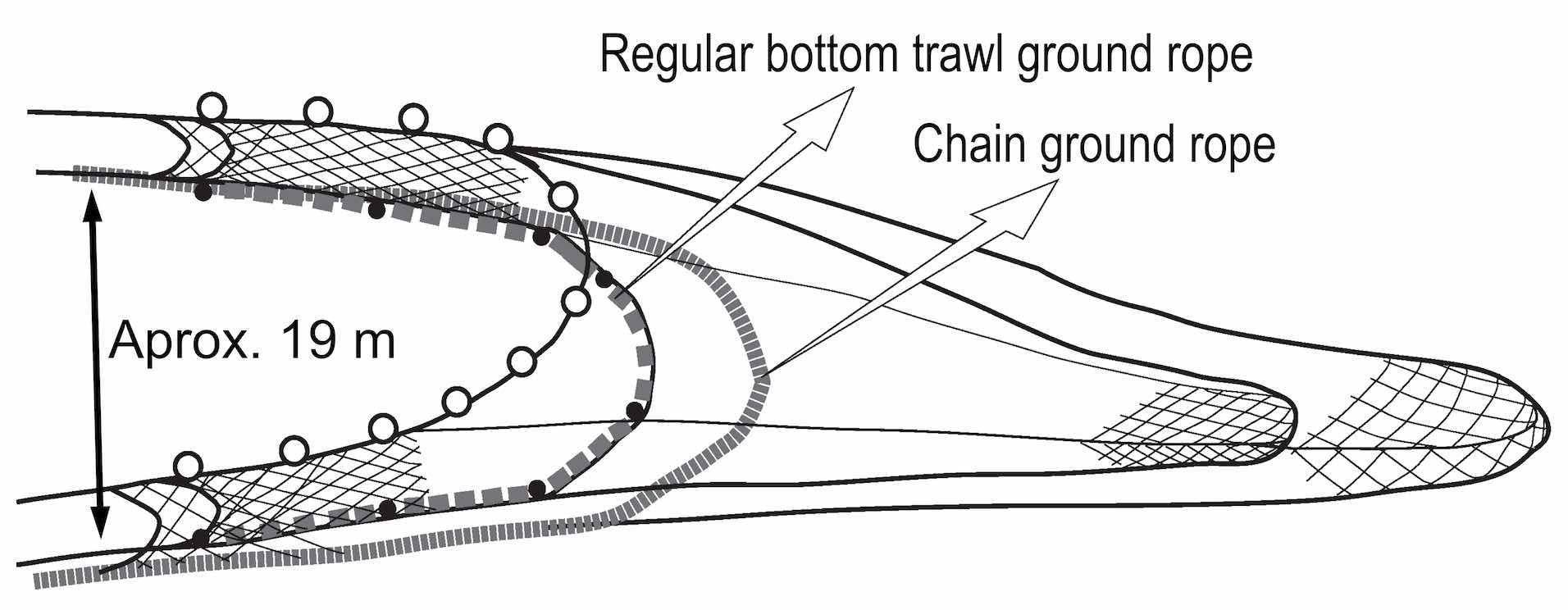


**Supplementary Fig. S6.** Schematic diagram of the net used for the “Extra net experiment” to estimate catch efficiency. The actual bottom trawl net with a regular ground rope is wrapped with an outer experimental cover net of 8 mm mesh size and a chain ground rope.

**Supplementary Table S2.** Catch efficiency of the bottom trawl net for *S. horneri* estimated by the “Extra net experiment”. By using the wet weight of *S. horneri* debris caught by the inside bottom trawl net (*TW*) and that of debris caught by the outside extra cover net (*CW*) for each trawling operation, the catch efficiency *E* was calculated by *TW* / (*TW* + *CW*). *An asterisk indicates the average bottom trawl net catch efficiency for *S. horneri*, and ± *SD* indicates the standard deviation.

|  | Trawling location | | Bottom depth  (m) | Catch by the inside  bottom trawl net (*TW*)  (g wet weight) | Catch by the outside  cover net (*CW*)  (g wet weight) | Catch efficiency  E = *TW* / (*TW* + *CW*) |
| --- | --- | --- | --- | --- | --- | --- |
| 1 | 38.43 °N | 142.00 °E | 350 | 10 | 80 | 0.111 |
| 2 | 38.93 °N | 142.09 °E | 350 | 20 | 110 | 0.154 |
| 3 | 38.42 °N | 142.06 °E | 450 | 3 | 38 | 0.073 |
| 4 | 38.42 °N | 142.06 °E | 450 | 9 | 28 | 0.243 |
|  |  |  |  |  |  | * 0.15 ± 0.07 |

**Attributes of floating macrophyte particle tracking simulator**

Filippi and Bisgambiglia (2004) and Filippi *et al.* (2011) developed a particle tracking simulation code based on DEVS (discrete event systems specification) simulator core for oil spill and fire propagation modeling. This DEVS simulator core was also applied to study the transport of floating *S. horneri* detached from the coasts of eastern China, using Princeton Ocean Model (POM) velocity forcing data (Filippi *et al*., 2010). The results were validated against the observed data of floating *S. horneri* distributions in the East China Sea (Komatsu *et al*., 2007). Passive particles deployed off the coasts of eastern China were transported northeastward along the Kuroshio front, and this corresponded well to the observed *S. horneri* distributions and buoy trajectories. Based on those results, reasonably good agreement was confirmed with the DEVS particle tracking simulator core for predicting the transportation route of floating *S. horneri* from the coastal beds to offshore waters.

Filippi, J. B. & Bisgambiglia, P. JDEVS: an implementation of a DEVS based on formal framework for environmental modeling. *Concepts, Methods and Application in Environment Model Integration* **19**, 261–271 (2004).

Filippi, J. B., Morandini, F., Balbi, J. H. & Hill, D. R. C. Discrete Event Front-tracking Simulation of a Physical Fire-spread Model. *Simulation* **87**, 555–580 (2011).

Filippi, J. B., Komatsu, T. & Tanaka, K. Simulation of drifting seaweeds in East China Sea. *Ecological Informatics* **5**, 67–72 (2010).

Komatsu, T. *et al*. Distribution of drifting seaweeds in eastern East China Sea. *Journal of Marine Systems* **67**, 245–252 (2007).

**Practical procedure of the two-way PTM to estimate origins of sunken macrophytes**

The two-way PTM origin-estimating algorithm was conducted along with the above-mentioned floating macrophyte particle tracking simulator and the OFES sea-surface velocity forcing data. The simulation of floating macrophyte trajectory in positive time (called 'forward-in-time particle tracking') and in reverse time (called 'backward-in-time particle tracking') were conducted with this simulator to conduct the two-way PTM. The detail procedure conducted for the coastal origin estimation of *S. horneri* is summarized in a flow chart in Supplementary Fig. S7.

In the first step, 10,000 particles were released from the time and position of all *S. horneri* debris caught by the bottom trawl surveys in spring of 2008, 2009 and 2010 (Supplementary Fig. S8, **a1**, **b1**, **c1**). The particle positions were computed by the Lagrangian approach with backward forcing OFES velocity data loaded in reverse time (that is, the backward-in-time particle tracking). The locations of the coasts that the particles reached were regarded as candidate origins. The candidate origins were determined for each half-month interval of a time-reversing period such as between 1–14 days before (Supplementary Fig. S8, **a2**, **b2**, **c2**), 15–30 days before (Supplementary Fig. S8, **a3**, **b3**, **c3**), and 31–45 days before the collection date (Supplementary Fig. S8, **a4**, **b4**, **c4**). The figures illustrate that the candidate origins were located at the Pacific side of the Japan archipelago within 130−142°E and 30−40°N. We searched the candidate origins for two additional months of time reversing. However, the area of candidate origins was overextended because the backward-in-time particles that released from the *S. horneri* collection points tended to disperse endlessly according to the reversing-time without any reasonable limit. We thus investigated the candidate origins for only an above-mentioned 45-day period of time reversing. Then we used these candidate origins for the following procedure of the two-way PTM.

In the second step, 10,000 particles were again released from each candidate origin with forward forcing OFES velocity data loaded in chronological order (that is, the forward-in-time particle tracking). Particles were released from the mid-date of each categorized half-month interval determined by the above-mentioned backward-in-time particle tracking. The trajectories of these forward-in-time particles were then calculated until the date when the backward-in-time particle-tracking model was started (that is, the date when *S. horneri* were collected from the seafloor).

In the third step, based on the locations of the forward-in-time particles that were transported to offshore waters, we calculated the standard deviation ellipse of their spatial distribution with 2σ major and minor axes. The ellipse was then displayed on a longitudinal and latitudinal coordinate map. If the trawl station (that is, the position where the backward-in-time simulation started in the first step) was located within the standard deviation ellipse, the candidate origin coast where particles were released by the forward-in-time simulation in the second step was regarded as an origin coast (Fig. 7**a**–**c**).

The coast of origin of *S. horneri* collected in summer was estimated with the same procedure as described above. According to the estimated candidate origins (Supplementary Fig. S9), their origins were determined (Fig. 7**d**–**f**).


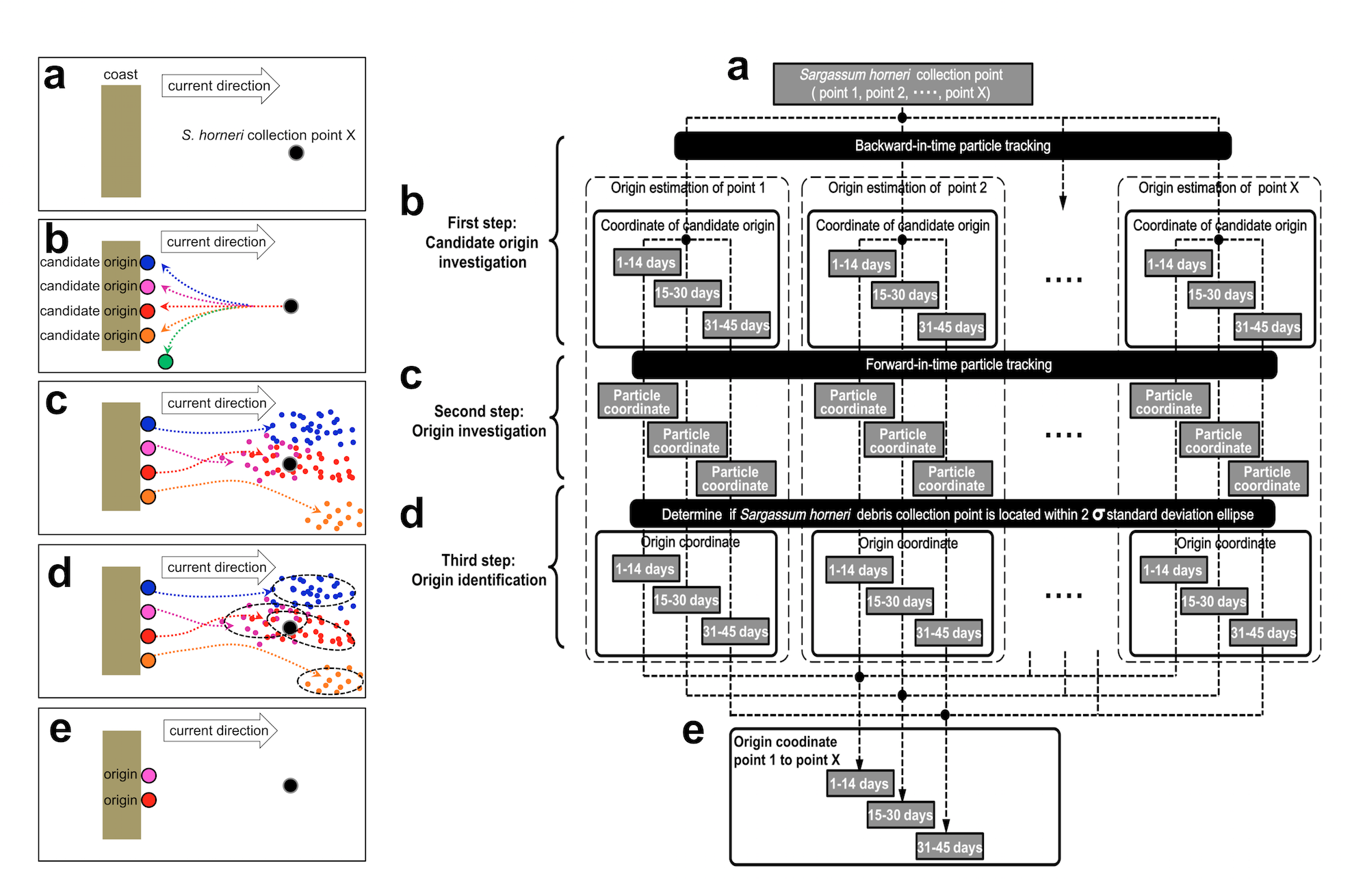


**Supplementary Fig. S7．**Flow chart of the two-way PTM used for identifying the origins of *S. horneri* debris. (**a**) Particles as drifting macrophyte rafts are released from a bottom-trawl station at a collection date with the backward-in-time particle tracking. (**b**) The positions where particles reached the coast are then regarded as candidate origins. (**c**) Particles are released again from each candidate origin with the forward-in-time particle tracking, and the positions of the particles are calculated until the collection date. (**d**) If the trawl survey station is located inside the 2σ standard deviation ellipse that is calculated using the final particle positions, (**e**) the candidate origins where those particles were released previously are regarded as an origin.

**
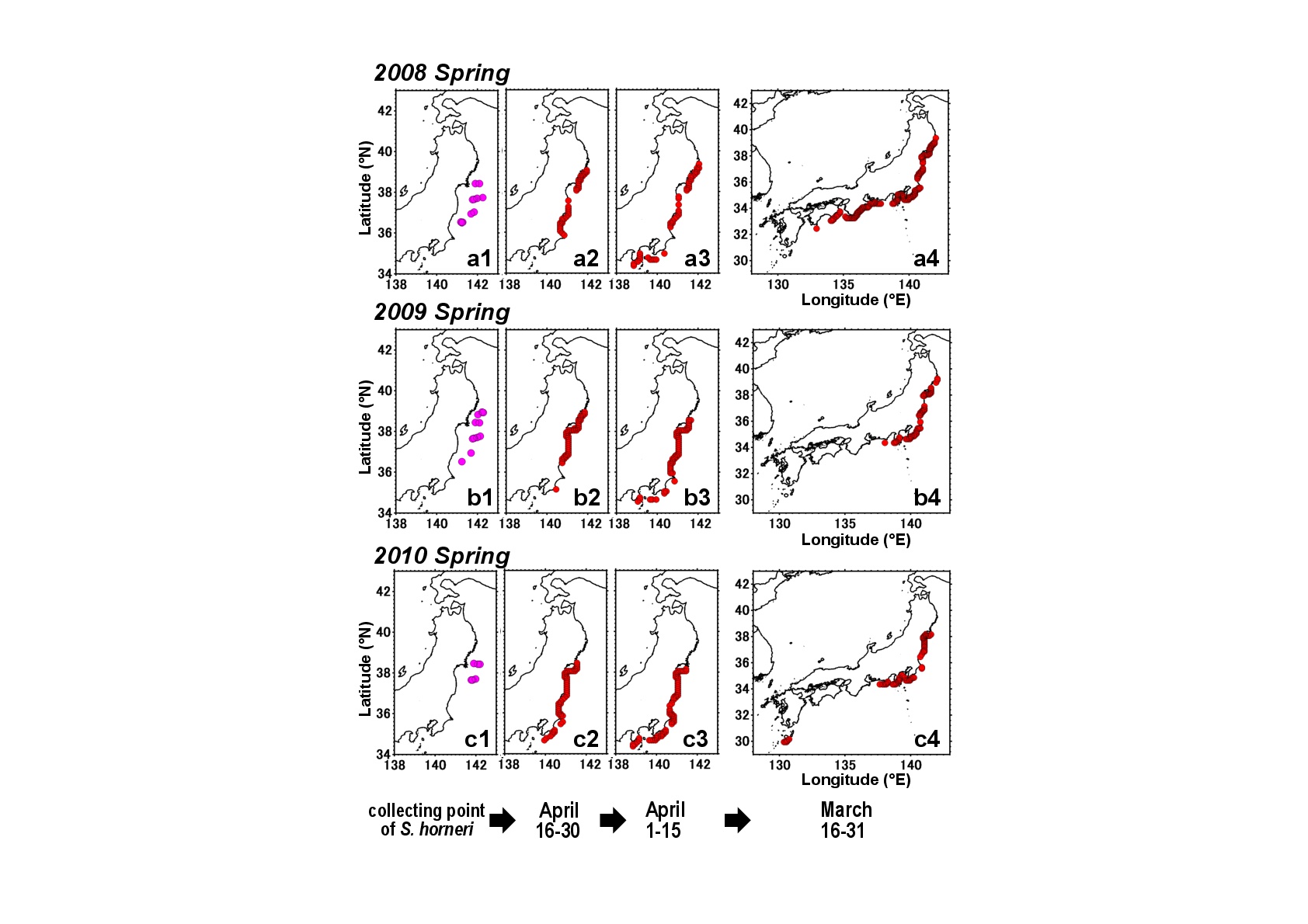
**

**Supplementary Fig. S8.** Candidate origins of *S. horneri* debris collected by bottom-trawl surveys in the spring of 2008 (upper panels, **a**), 2009 (middle panels, **b**) and 2010 (lower panels, **c**) estimated by the backward-in-time particle tracking. In column 1, particles of floating *S. horneri* rafts were released from the trawl stations (purple dots) where *S. horneri* debris was collected. Columns 2, 3 and 4 from the left are the positions of candidate origins (red dots), for those 1–14 days before, 15–30 days before, 31–45 days before their date of collection by the bottom trawling, respectively. The maps were generated using Matlab R2011b (https://www.mathworks.com/products/matlab).


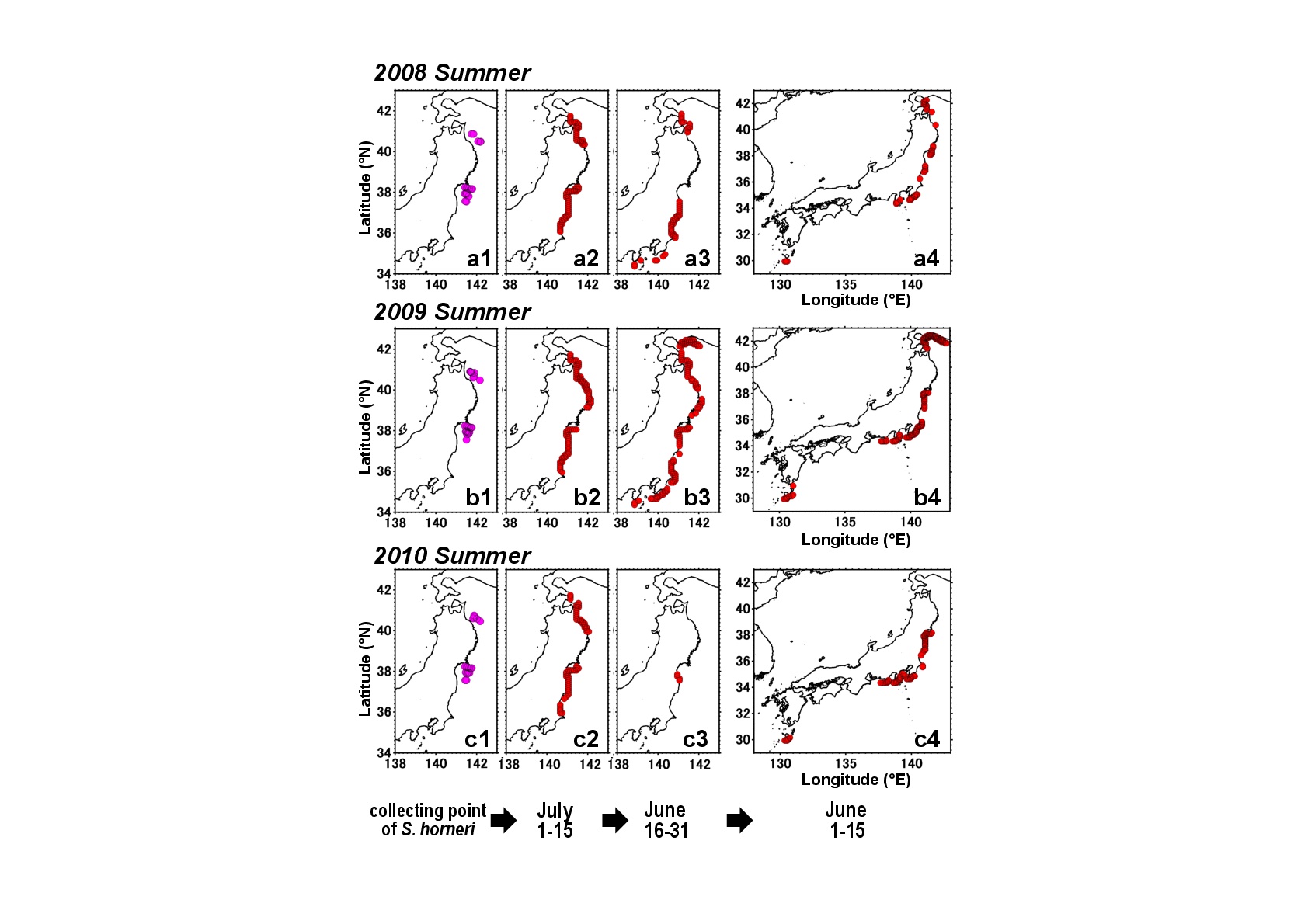


**Supplementary Fig. S9.** Candidate origins of *S. horneri* debris collected by bottom-trawl surveys in the summer of 2008 (upper panels, **a**), 2009 (middle panels, **b**) and 2010 (lower panels, **c**) estimated by the backward-in-time particle tracking. In column 1, particles of floating *S. horneri* rafts were released from the trawl stations (purple dots) where *S. horneri* debris was collected. Columns 2, 3 and 4 from the left are the positions of candidate origins (red dots), for those 1–14 days before, 15–30 days before, 31–45 days before their date of collection by the bottom trawling, respectively. The maps were generated using Matlab R2011b (https://www.mathworks.com/products/matlab).
